# Efficacy of ultrasound-accelerated versus traditional catheter-directed thrombolysis in treatment of lower extremity deep venous thrombosis: A protocol for systematic review and meta-analysis

**DOI:** 10.1097/MD.0000000000026454

**Published:** 2021-07-09

**Authors:** Shan Ma, Zhizhen Zhao, Zhijun Song, Li Wang

**Affiliations:** a Department of Interventional Medicine, Zhangye Second People's Hospital, Zhangye City, Gansu, China; b Department of Pharmacy, Zhangye Second People's Hospital, Zhangye City, Gansu, China; c Department of Five Sense Organs, Zhang Ye People's Hospital Affiliated To Hexi University, Zhangye City, Gansu, China.

**Keywords:** deep vein thrombosis, meta-analysis, protocol, systematic review, ultrasonic-accelerated thrombolysis

## Abstract

**Background:**

There is no meta-analysis or review in the literature to compare and evaluate the difference and effectiveness of ultrasonic-accelerated thrombolysis (UAT) and catheter directed thrombolysis (CDT) in lower extremity deep vein thrombosis (DVT) patients. Therefore, we conducted this protocol of systematic review and meta-analysis to evaluate the efficacy between UAT and CDT for patients with lower extremity DVT.

**Methods:**

We followed the Preferred Reporting Items for Systematic Reviews and Meta-Analyses Protocols reporting guidelines to conduct this study. Reviewers will search the PubMed, Cochrane Library, Web of Science, and EMBASE online databases using the key phrases “deep venous thrombosis,” “thrombolysis,” and “ultrasound-accelerated” for all cohort studies published up to July 22, 2021. There is no restriction in the dates of publication or language in the search for the current review. The primary outcome is major bleeding. Secondary outcomes include health-related quality of life and complications such as recurrent venous thromboembolism, pulmonary embolism, in-stent thrombosis, and death. Review Manager software (v 5.4; Cochrane Collaboration) will be used for the meta-analysis. A *P* value of < .05 is considered to be statistically significant.

**Results:**

We hypothesized that these two methods would provide similar therapeutic benefits.

**OSF registration number:**

10.17605/OSF.IO/YZB3H.

## 1 Introduction

Deep vein thrombosis (DVT) is a serious disorder with a lifetime incidence of 2.5 to 5.0 percent, with a long-term complication known as post-thrombotic syndrome persists in 40 to 60 percent of patients. Standard treatment for DVT includes immediate anticoagulant therapy to prevent thrombus growth and embolism, as well as early activity and compression therapy, which may reduce residual thrombotic load and the occurrence of post-thrombotic syndrome.^[1,2]^ Although effective for the majority of patients, this treatment is inadequate for those at highest risk for post-thrombotic syndrome, especially those with iliofemoral thrombosis.^[3]^

In order to prevent this complication and its significant medical, social, and economic consequences, a variety of strategies for early thrombus clearance have emerged. Catheter directed thrombolysis (CDT) in combination with percutaneous mechanical thrombectomy is becoming increasingly important due to its effectiveness in achieving venous patency and preventing secondary venous insufficiency. However, there are still concerns toward treatment time and risk of bleeding complications.^[4,5]^

Ultrasonic-accelerated thrombolysis (UAT) is a novel approach in which a thrombolytic agent is delivered via an infusion pump while ultrasonic energy is applied to the luminal thrombus, whereas traditional CDT uses only a catheter to deliver fibrinolytic drugs through multiple lateral holes.^[6]^ In vitro research has shown that the ultrasound waves influence the fibrin strands and increase uptake of thrombolytic drug in the thrombus.^[7]^ Moreover, several previous studies have compared and assessed the lysis results between UAT and CDT, but with different conclusions.^[8–10]^ As far as we know, there is no meta-analysis or review in the literature to compare and evaluate the difference and effectiveness of the two methods in lower extremity DVT patients. Therefore, we conducted this protocol of systematic review and meta-analysis to evaluate the efficacy between UAT and CDT for patients with lower extremity DVT.

## 2 Materials and methods

### 2.1 Search strategy

We followed the Preferred Reporting Items for Systematic Reviews and Meta-Analyses Protocols reporting guidelines to conduct this study. The prospective registration has been approved by the Open Science Framework registries (with the number 10.17605/OSF.IO/YZB3H). Reviewers will search the PubMed, Cochrane Library, Web of Science, and EMBASE online databases using the key phrases “deep venous thrombosis,” “thrombolysis,” and “ultrasound-accelerated” for all cohort studies published up to July 22, 2021. There is no restriction in the dates of publication or language in the search for the current review, and thus publication and language bias can be minimized. Ethical approval is not necessary because the present meta-analysis will be performed based on previous published studies.

### 2.2 Inclusion and exclusion criteria

Included studies are considered eligible if they met the Population, Intervention, Comparator, Outcomes, and Study design criteria as follows:

1. Population: lower extremity DVT;
2. Intervention: group with UAT;
3. Comparator: group with CDT;
4. Outcomes: the primary outcome is major bleeding. Secondary outcomes include health-related quality of life and complications such as recurrent venous thromboembolism, pulmonary embolism, in-stent thrombosis, and death.
5. Study design: cohort study.

Exclusion criteria include conference abstract, letters, review articles, studies with a sample size < 50, and studies with insufficient outcome data.

### 2.3 Study selection

Two independent authors will follow the unified search strategy to screen the titles and abstracts of potentially relevant studies. Any inconsistencies between reviewers will be resolved through discussion and consensus. If a consensus cannot be reached, a senior author will be consulted for a final decision.

### 2.4 Data extraction

Two independent authors will extract the following descriptive raw information from the selected studies: study characteristics such as author, study design, study language, publication year, mean follow-up period; patient demographic details such as number, average age, body mass index and gender ratio; details of interventions, and outcome measures. The primary outcome is major bleeding. Secondary outcomes include health-related quality of life and complications such as recurrent venous thromboembolism, pulmonary embolism, in-stent thrombosis, and death. If the data are missing or cannot be extracted directly, we will contact the corresponding authors to ensure that the information integrated. Otherwise, we calculate them with the guideline of Cochrane Handbook for Systematic Reviews of Interventions 5.1.0.

### 2.5 Methodological quality assessment

The quality of randomized trials will be assessed by Cochrane risk of bias tool for randomized controlled trials and the risk of bias in Non-Randomized Studies of Interventions for non-randomized, observational studies. Each paper will be reviewed by one reviewer and verified by a second and disagreements will be resolved by discussion with a third reviewer. A meta-analysis will be conducted when 3 or more trials reported an outcome of interest. We also will perform the sensitivity analysis to evaluate whether the differences of study design had an impact on the overall estimate and data. Review Manager software (v 5.4; Cochrane Collaboration) will be conducted for statistical investigation and a funnel plot analysis will be drawn to assess the publication bias if there are more than 10 studies included.

### 2.6 Data analysis

Review Manager software (v 5.4; Cochrane Collaboration) will be used for the meta-analysis. Continuous variables are extracted and analyzed to mean value ± SD. Standardized mean differences with a 95% confidence interval are assessed for continuous outcomes. The heterogeneity is assessed by using the Q test and I^2^ statistic. An I^2^ value of < 25% is chosen to represent low heterogeneity and an I^2^ value of > 75% to indicate high heterogeneity. All outcomes are pooled on random-effect model. A *P* value of < .05 is considered to be statistically significant.

## 3 Discussion

UAT is a novel approach in which a thrombolytic agent is delivered via an infusion pump while ultrasonic energy is applied to the luminal thrombus, whereas traditional CDT uses only a catheter to deliver fibrinolytic drugs through multiple lateral holes.^[6]^ In vitro research has shown that the ultrasound waves influence the fibrin strands and increase uptake of thrombolytic drug in the thrombus.^[7]^ Moreover, several previous studies have compared and assessed the lysis results between UAT and CDT, but with different conclusions.^[8–10]^ As far as we know, there is no meta-analysis or review in the literature to compare and evaluate the difference and effectiveness of the two methods in lower extremity DVT patients. Therefore, we conducted this protocol of systematic review and meta-analysis to evaluate the efficacy between UAT and CDT for patients with lower extremity DVT.

## Author contributions

**Conceptualization:** Zhijun Song.

**Data curation:** Shan Ma, Zhizhen Zhao.

**Formal analysis:** Shan Ma, Zhizhen Zhao.

**Funding acquisition:** Li Wang.

**Investigation:** Shan Ma, Zhizhen Zhao.

**Methodology:** Zhizhen Zhao, Zhijun Song.

**Project administration:** Li Wang.

**Resources:** Li Wang.

**Software:** Shan Ma.

**Supervision:** Li Wang.

**Validation:** Zhijun Song.

**Visualization:** Zhijun Song.

**Writing – original draft:** Shan Ma.

**Writing – review & editing:** Li Wang.
